# Cyclic mechanical stretch up-regulates hepatoma-derived growth factor expression in cultured rat aortic smooth muscle cells

**DOI:** 10.1042/BSR20171398

**Published:** 2018-03-16

**Authors:** Ying-Hsien Kao, Po-Han Chen, Cheuk-Kwan Sun, Yo-Chen Chang, Yu-Chun Lin, Ming-Shian Tsai, Po-Huang Lee, Cheng-I Cheng

**Affiliations:** 1 Department of Medical Research, E-Da Hospital, Kaohsiung, Taiwan; 2 Department of Ophthalmology, Kaohsiung Medical University Hospital, Kaohsiung Medical University, Kaohsiung, Taiwan; 3 Department of Surgery, E-Da Hospital, Kaohsiung, Taiwan; 4 Division of Cardiology, Department of Internal Medicine, Kaohsiung Chang Gung Memorial Hospital, Kaohsiung, Taiwan

**Keywords:** hepatoma-derived growth factor, mechanical stretch, signal transduction, vascular smooth muscle, vascular injury

## Abstract

Hepatoma-derived growth factor (HDGF) is a potent mitogen for vascular smooth muscle cells (SMCs) during embryogenesis and injury repair of vessel walls. Whether mechanical stimuli modulate HDGF expression remains unknown. The present study aimed at investigating whether cyclic mechanical stretch plays a regulatory role in HDGF expression and regenerative cytokine production in aortic SMCs. A SMC cell line was grown on a silicone-based elastomer chamber with extracellular matrix coatings (either type I collagen or fibronectin) and received cyclic and uniaxial mechanical stretches with 10% deformation at frequency 1 Hz. Morphological observation showed that fibronectin coating provided better cell adhesion and spreading and that consecutive 6 h of cyclic mechanical stretch remarkably induced reorientation and realignment of SMCs. Western blotting detection demonstrated that continuous mechanical stimuli elicited up-regulation of HDGF and proliferative cell nuclear antigen, a cell proliferative marker. Signal kinetic profiling study indicated that cyclic mechanical stretch induced signaling activity in RhoA/ROCK and PI3K/Akt cascades. Kinase inhibition study further showed that blockade of PI3K activity suppressed the stretch-induced tumor necrosis factor-α (TNF-α), whereas RhoA/ROCK inhibition significantly blunted the interleukin-6 (IL-6) production and HDGF overexpression. Moreover, siRNA-mediated *HDGF* gene silencing significantly suppressed constitutive expression of IL-6, but not TNF-α, in SMCs. These findings support the role of HDGF in maintaining vascular expression of IL-6, which has been regarded a crucial regenerative factor for acute vascular injury. In conclusion, cyclic mechanical stretch may maintain constitutive expression of HDGF in vascular walls and be regarded an important biophysical regulator in vascular regeneration.

## Introduction

Vascular smooth muscle cells (SMCs) are not terminally differentiated and capable of undergoing marked changes in phenotype in response to changes in local environmental cues that normally control its differentiation and maturation. The differentiation of SMCs is a strictly regulated process that directs the cells to express a unique repertoire of contractile intracellular cytoskeleton proteins, ion channels, membrane receptors, and signaling molecules necessary for its contractile phenotype [1]. Under pathogenic conditions such as vessel injury, fully differentiated medial SMCs undergo a process referred to as phenotypic modulation, characterized by decreased expression of markers of differentiated SMCs, such as smooth muscle α-actin (α-SMA). The accelerated SMC growth and increased synthesis of extracellular matrix (ECM) are two hallmarks for the damaged vessel under repair [1]. In the context of phenotypic modulation, mechanical stretch has long been claimed to be one of the critical factors regulating SMC morphological alteration, differentiation, and physiological function [2,3]. Earlier *ex vivo* studies have reported that cyclic and uniaxial mechanical stretch induces reorienting and aligning effect prominently noted in cultured SMCs derived from human bladder [4] and airway [5] as well as from aortic tissues of animals [6,7]. In addition, the mechanical stretch increases expression of SMC-specific marker smooth muscle myosin heavy chain, but decreases expression of nonmuscle type myosin [8]. In the context of induction of signal transduction, mechanical stretch is found to stimulate SMC growth through activating multiple signaling pathways including phosphatidylinositol 3-kinase (PI3K)/Akt [9,10], Rho kinase [11], and mitogen activated protein kinase (MAPK) cascades [12–16]. These findings collectively support that biophysical factors like mechanical stress play an important role in control of vascular SMC differentiation in a manner similar to that in skeletal and cardiac muscles.

Hepatoma-derived growth factor (HDGF) is a ubiquitous growth factor originally isolated from conditioned medium of Huh-7 hepatoma cells [17,18]. Earlier studies identified that HDGF is functionally involved in development of many organs [17,19]. The up-regulated HDGF in both human carotid and balloon-injured rat carotid arteries is found to colocalize with α-SMA and proliferative cell nuclear antigen (PCNA) and exert mitogenic and motogenic effects on vascular SMCs during vascular injury [20–22]. Our and other groups’ findings indicate that HDGF plays pleiotropic roles in regulation of hepatic fibrogenesis [23], and stimulates angiogenesis through increasing vascular endothelial growth factor expression and endothelial cell proliferation [24]. The proangiogenic function of HDGF has been linked to cancer development [25,26] and metastasis [27]. All these evidence suggests that HDGF expression is pathophysiologically associated with vascular development and involved in repairing mechanism of diseased vascular walls. However, what factor precisely regulates the HDGF expression in vascular SMCs remains unclear to date. The present study aimed to determine whether *in vitro* cyclic mechanical stretches modulate HDGF expression in cultured rat aortic SMCs. We demonstrated that cyclic uniaxial mechanical stretching not only exerted morphological reorientation, but also up-regulated signaling activation of PI3K/Akt and RhoA/ROCK pathways as well as increased expression of HDGF and proinflammatory cytokines in cultured rat aortic SMCs.

## Materials and methods

### Cell culture

An immortalized cell line of rat aortic SMCs (clone HEP-SA) purchased from Bioresource Collection and Research Center (BCRC no. 60523, Food Industry Research and Development Institute, Hsinchu, Taiwan) were cultured in DMEM medium supplemented with 10% FBS, 2 mM L-glutamine, 100 I.U./ml penicillin, 100 μg/ml streptomycin, 250 ng/ml amphotercin (Gibco/Invitrogen, Gaithersburg, MD), and 0.2 mg/ml G418 (A.G. Scientific, San Diego, CA) in a humidified atmosphere containing 5% CO_2_ at 37°C.

### ECM coating

Collagen stock solution (5 mg/ml) was extracted from rat tail tendons as previously described [28]. Fibronectin (FN) stock solution (1 mg/ml) was purchased from Sigma-Aldrich (St. Louis, MO). Prediluted ECMs in ice-cold PBS at indicated concentrations (1–25 μg/ml) were added into silicone chambers. Three milliliters solution for each three-well chamber (7.5 cm^2^) and 10 ml for one-well chamber (25 cm^2^). Silicone elastomer chambers were sealed, incubated overnight at 4°C, and rinsed three times with PBS before cell seeding.

### Cyclic mechanical stretching treatment

**Figure 1:**
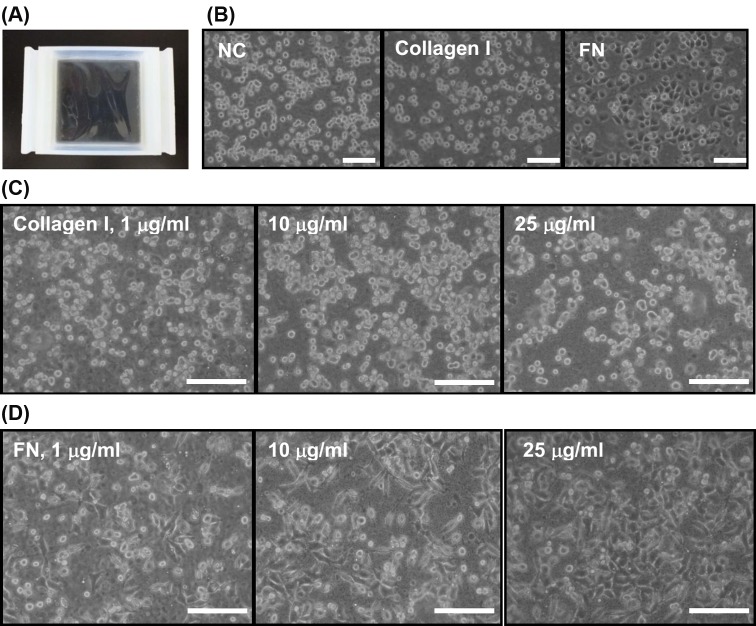
Silicone elastomer chamber and optimization of extracellular matrix (ECM) coating by morphological observation (**A**) A top view of the silicone-based elastomer chamber. (**B**) Morphology of rat aortic smooth muscle cells (SMCs) seeded on noncoated (NC), type I collagen, and fibronectin (FN)-coated and silicone chambers. ECM precoating was performed by incubation with either PBS, 25 μg/ml of type I collagen, or 10 μg/ml of FN at 24 h before seeding. Microphotographs were taken at 60 min after seeding. Optimization of ECM coating dose was determined by seeding 8 × 10^5^ of SMCs onto the silicone chambers precoated with indicated doses of type I collagen (**C**) or FN (**D**). Cell morphology observed at 60 min after seeding showed that FN exerted better cell spreading effect on SMCs; scale bars = 20 μm.

To assess mechanical cyclic stretch, 80% confluent SMCs were trypsinized, counted, and seeded on silicone elastomer-bottomed and collagen- or FN-coated chambers. A photograph of silicone chamber is shown in Figure 1A. After 2 h of incubation for cell spreading, the silicone chamber was applied onto the mechanical stretch system using a programmable cyclic stretching instrument (ATMS Boxer^TM^, TAIHOYA Corporation, Taiwan) that generates defined uniaxial and cyclic longitudinal deformation at 60 cycles/min (1 Hz) by 10 % elongation on silicone-based chamber. Accuracy of device-induced elastomer deformation was previously verified by morphometric measurement (Supplementary Figure S1). All procedures were carried out in a humidified CO_2_ incubator at 37°C.

### Morphological observation and morphometrics

SMCs grown in silicone elastomer chambers before and during mechanical stretching were subjected to microscopic observation under an inverted phase-contrast microscope (Axiovert® 200, Carl Zeiss, Gottingen, Germany) and documented with a digital image documentation system (AxioVision® software, Carl Zeiss). The angles between SMC long axis and stretch direction were measured by using ImageJ software (NIH). The cell axial line parallel to the stretch direction was defined as zero degree. At least 200 of cells were analyzed and the mean angles of all counted events are shown in representative figure.

### Fluorescent visualization of intracellular F-actin

To visualize cellular distribution of F-actin, the adherent SMCs grown on transparent silicone elastomer before and after stretching were washed with prewarmed PBS, fixed in 3.7% formaldehyde solution for 10 min, and permeabilized with 0.1% Triton X-100 in PBS for 5 min at room temperature. The cells were then incubated with 5–10 units of fluorescein (FITC)-labeled phalloidin (Invitrogen, Eugene, OR) for 30 min at 4°C. After washes with PBS, the nuclei were counterstained with DAPI, and the slips were mounted and observed under a fluorescent microscope (AxioPlan®, Carl Zeiss).

### Protein extraction and Western blotting

Total protein of cultured SMCs were extracted by lysing the cells in cold RIPA buffer (50 mM Tris-HCl, pH 7.4, 5 mM EDTA, 1% Triton X-100, 0.4% sodium cacodylate, and 150 mM NaCl) in the presence of protease and phosphatase inhibitors (Roche, Molecular Biochemicals, Mannheim, Germany). After centrifugation to remove cell debris, supernatants were subjected to SDS/PAGE, using 8% or 12% acrylamide gels under reducing conditions. Proteins were subsequently electrotransferred onto a nitrocellulose membrane (Sartorius AG, Göttingen, Germany) following conventional protocols. Blots were blocked in 5% skimmed milk/PBS with 0.1% Tween-20 (PBS-T; 100 mM phosphate buffer, 137 mM NaC1, 0.1% Tween-20, pH7.4) for 1 h at room temperature, followed by overnight incubation with primary antibodies at 4°C. The primary antibodies were diluted in PBS-T with 5% skimmed milk. All antibodies raised against phosphorylated type of Akt (Ser473), ERK1/2 (Thr202/Tyr204), JNK (Thr183/Tyr185), and p38 MAPK (Thr180/Tyr182) were purchased from Cell Signaling (Danvers, MA). Anti-HDGF and anti-phosphorylated myosin-binding subunit of myosin light chain phosphatase (p-MBS, phosphorylated at Thr853) antibodies were obtained from Abcam (Cambridge, MA). Anti-PCNA antibody was from BD Biosciences (San Jose, CA). After five washes in PBS-T, the blots were incubated with secondary antibodies (horseradish peroxidase coupled with anti-mouse or anti-goat IgG) at 1:4000 dilution. The enhanced chemiluminescence (ECL, Millipore, Piscataway, NJ) detection kit was used to visualize the immunoreactive protein on the blot according to the manufacturer’s instructions. The illuminant signal were recorded on a digital imaging system and analyzed in a densitometrical analysis system (BioSpectrum, Ultra-Violet Products Ltd., Cambridge, U.K.). The relative protein levels were expressed as the density ratios of interested protein to internal Actin contents in the same specimen, and the negative control was taken as 1.0.

### Kinase inhibitors and treatment

Selective kinase inhibitors, including wortmannin for PI3K and fasudil for rhoA/ROCK inhibition, were purchased from Sigma-Aldrich Company. Chemical inhibitors were all dissolved in dimethyl sulfoxide (DMSO) at 10 mM and added into culture medium at 10 μM (except fasudil at 30 μM) at 1 h prior to mechanical stretching procedures.

### ELISA detection

Conditioned media from cultured cells were collected at the indicated time points. The soluble cytokines including tumor necrosis factor-α (TNF-α) and interleukin-6 (IL-6) were measured by using commercially available ELISA detection kits (Biolegend, San Diego, CA) according to the manufacturer’s instructions.

### RNA interference of *HDGF*

To determine the role of HDGF in constitutive production of cytokines in SMCs, cells were transfected with either small interfering RNA (siRNA) against *HDGF* gene or scramble RNA control at 100 nM (Ambion/Invitrogen, Carlsbad, CA) by Lipofectamine 2000 (Invitrogen, Grand Island, NY). After the silencing efficiency being verified at 48 h post siRNA transfection by Western blot, supernatants of *HDGF* gene-silenced cells were collected for ELISA.

### Statistical analysis

All density data are expressed as mean ± standard error of the mean (SEM). Comparisons among groups are analyzed by using unpaired Student’s *t*-test with two-tailed analysis, followed by Bonferroni post hoc tests. Statistical significance between groups is declared when *P*<0.05.

## Results

### Biocompatibility of silicone elastomer chamber and optimization of ECM coating

To determine the biocompatibility of silicone elastomer in a rat aortic SMC line, the cells were seeded onto uncoated, type I collagen-, or FN-coated surfaces. The morphology of cell adhesion and spreading was documented through an inverted microscope. Microscopic morphology observation at 3 h post seeding clearly showed that the FN-coated elastomer chamber generated the best adhesion-promoting effect in that almost all seeded cells adhered to substratum and showed spreading morphology, when compared with those in noncoated and type I collagen-coated groups (Figure 1B). To optimize the ECM coating dose, aortic SMCs were seeded onto elastomer chamber precoated with different doses of type I collagen or FN. Microscopic morphology showed that the elastomer coated with type I collagen merely provides cell-to-matrix adhesion, but not remarkably improves spreading of adherent SMCs (Figure 1C). By contrast, FN-coated elastomer surface enhanced both adhesion and spreading capacity of aortic SMCs in a dose-dependent manner (Figure 1D). Optimal coating doses for type I collagen and FN were, respectively, set at 25 and 10 μg/ml for economic purpose.

### Morphological alterations of rat aortic SMCs induced by cyclic mechanical stretch

**Figure 2:**
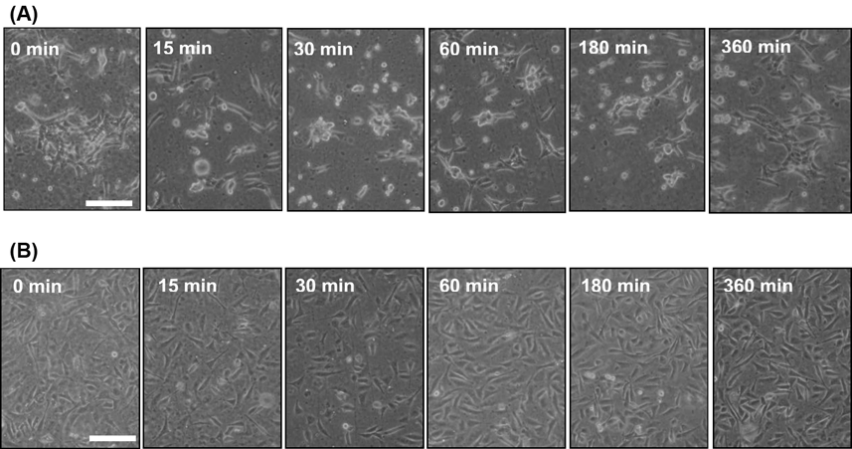
Time course morphology observation of rat aortic smooth muscle cells (SMCs) grown on different coatings and receiving cyclic mechanical stretches Silicone elastomer chambers were precoated with type I collagen at 25 μg/ml (**A**) or fibronectin at 10 μg/ml (**B**) for overnight incubation at 4 °C. About 8 × 10^5^ of SMCs were then seeded onto silicone chambers for 3 h till cellular attachment and spreading. The morphology was observed and documented after the cells received uniaxial and cyclic 10% deformation at constant frequency (1 Hz) for the indicated consecutive durations; scale bars = 20 μm.

To further observe the morphological alteration in the cells receiving cyclic mechanical stretch, cultured aortic SMCs were seeded onto either type I collagen- or FN-coated silicone elastomer chambers at 3 h before stretching. The chambers were mounted on mechanical stretch-generating machine equipped in a CO_2_ incubator and consecutively received uniaxial (longitudinal) and cyclic 10% deformation at constant 1 Hz frequency. Morphology observation under an inverted phase-contrast microscope indicated that the spindle-shaped SMCs grown on collagen became rounded and remained adherent after being stretched for 15 min to 180 min (Figure 2A), suggesting that the biomechanical force may decrease cell-to-ECM affinity. After 6 h of consecutive stretching, the rounded cells started to respread and showed spindle-shape morphology, very likely due to restoration of the cell-to-ECM affinity. By contrast, the morphological effect of cyclic mechanical stretch was not prominently noted in the aortic SMCs grown on FN-coated elastomer surfaces (Figure 2B).

### Cyclic mechanical stretch-induced reorientation and cytoskeletal redistribution of rat aortic SMCs

**Figure 3:**
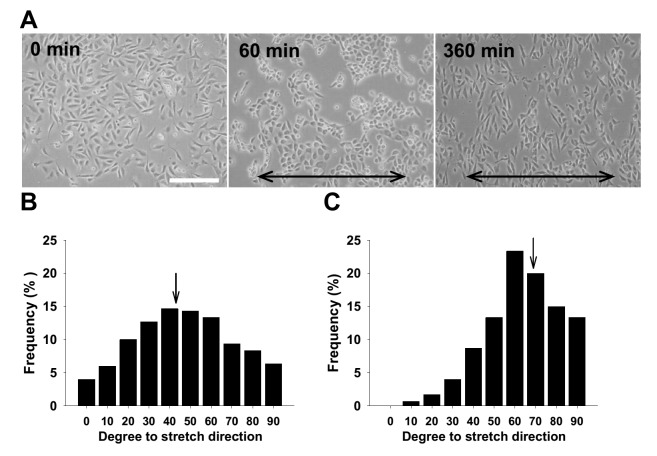
Reorientation and alignment of rat aortic smooth muscle cells (SMCs) induced by cyclic mechanical stretch (**A**) About 8 × 10^5^ of SMCs were seeded onto fibronectin-coated (at 10 μg/ml) silicone elastomer chambers for 3 h till cellular attachment and spreading, followed by consecutively uniaxial and cyclic 10% deformation at constant frequency (1 Hz). The morphology was observed and documented under inverted microscope at 0, 60, and 360 min. The lines with arrows on both ends indicate stretching direction; scale bars = 20 μm. Morphometrics was used to measure the angles between SMC long axes and stretching direction. Representative angle histograms of SMCs before (**B**) and after 360 min stretching (**C**) are shown. Arrows indicate the mean angle locations, 42.5 and 67.9 degrees respectively.

**Figure 4:**
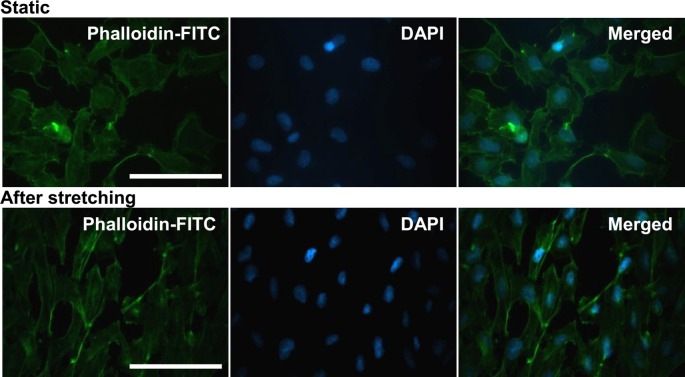
Effect of cyclic mechanical stretch on distribution of F-actin cytoskeleton in rat aortic smooth muscle cells (SMCs) About 8 × 10^5^ of SMCs were seeded onto fibronectin-coated (at 10 μg/ml) silicone elastomer chambers and received uniaxial and cyclic 10% stretches at constant frequency (1 Hz). The cells of static control (upper panel) and those receiving 360 min of mechanical stretches (lower panel) were subjected to FITC–phalloidin staining for intracellular distribution of F-actin in SMCs. DAPI was used to simultaneously visualize cell nuclei; scale bars = 20 μm.

Since a mechanical stretch-induced aligning effect has been reportedly noted in human bladder [4] and airway [5] SMCs as well as bovine- [6] and rat-derived [7] aortic SMCs, we next determined whether this effect could also be reproducibly seen in this cell line of rat aortic SMCs. The microphotographs taken during the given time frame confirmed that rat aortic SMCs also lost their characteristic spindle shape after 60 min of cyclic stretches, and the bipolar shape reappeared after 360 consecutive min (Figure 3A). Subsequent morphometrical measurement on the angles between SMC long axes and stretching direction demonstrated that the mean angle of the SMCs receiving cyclic stretches shifted toward 90 degree, perpendicular to the stretch direction (Figure 3B,C). To study the effect of strain-induced cytoskeletal reorganization in rat aortic SMCs, the static and stretched cells were subjected to F-actin visualization by FITC-phalloidin staining. The fluorescent staining results indicated that intracellular F-actin was indeed redistributed by cyclic mechanical stretch, showing intensified membranous localization (Figure 4).

### Cyclic mechanical stretch-induced HDGF up-regulation and proliferation in rat aortic SMCs

**Figure 5:**
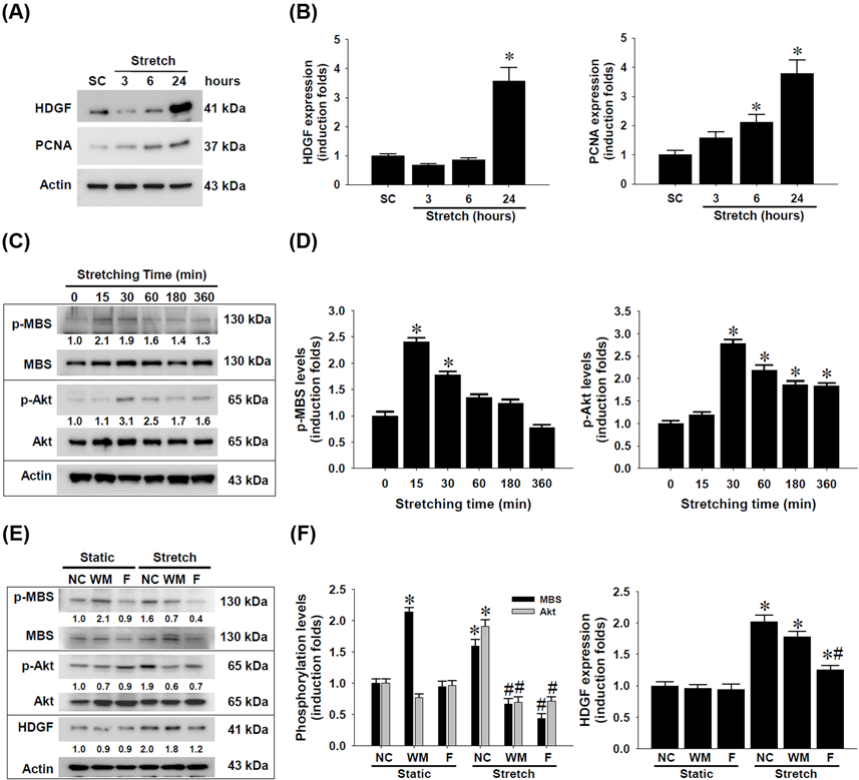
Up-regulation of HDGF expression in rat aortic smooth muscle cells (SMCs) by cyclic mechanical stretch and the signaling activity involved SMCs (8 × 10^5^ cells per chamber) were seeded on fibronectin-coated silicone elastomer chambers and received uniaxial and cyclic 10% stretches at constant frequency (1 Hz). (**A**) Protein lysates of SMCs receiving cyclic mechanical stretch for indicated time and static control (SC) collected at 24 h were subjected to Western blotting detection for HDGF and proliferative cell nuclear antigen (PCNA) as a proliferative marker. (**B**) Densitometric analysis of HDGF and PCNA expression levels. (**C**) SMCs grown on fibronectin-coated elastomer chambers received mechanical stretch at the indicated time and the protein lysates were subjected to Western blot detection of MBS and Akt phosphorylation levels. (**D**) Densitometric analysis of MBS and Akt phosphorylation levels. (**E**) Crosstalk of RhoA/ROCK and PI3K/Akt signaling cascades and their involvement in cyclic mechanical stretch-induced HDGF up-regulation in SMCs. The cells were treated with wortmannin (WM) or fasudil (**F**) at 10 μM for 1 h and received uniaxial and cyclic 10% stretches at frequency (1 Hz) for 1 h. The protein lysates were subjected to Western blot detection of HDGF and phosphorylation levels of MBS and Akt proteins. (**F**) Densitometric analysis of MBS and Akt phosphorylation as well as HDGF expression levels. The representative blot images obtained from three independent experiments are shown with induction folds compared with time zero or static negative control (NC). Density data are shown in mean ± SEM; *P<0.05 compared with SC or time zero control; ^#^*P*<0.05 compared with corresponding stretched NC.

For HDGF has long been identified as a SMC mitogen during vascular development [20] and injury [21,22,29], we next determined whether cyclic mechanical stretch modulates HDGF expression in rat aortic SMCs. Western blotting detection demonstrated that cyclic mechanical stretch remarkably up-regulated HDGF expression in SMCs compared with static control (Figure 5A,B). Along with HDGF up-regulation, cyclic mechanical stretch concomitantly increased expression of PCNA, a cellular marker for proliferation, strongly suggesting the contribution of endogenous HDGF expression to SMC proliferation.

### Cyclic mechanical stretch-activated PI3K and RhoA/ROCK signal transduction in rat aortic SMCs

Because a plethora of studies have reported the involvement of multiple signaling pathways in the behavioral changes of vascular SMCs by mechanical strain, including PI3K/Akt [9,30], JNK [31], ERK1/2 [32], and p38 MAPK [33], and that RhoA/ROCK signaling is responsible for the mechanical stress-triggered activation of MAPK pathways in different types of SMCs [33–37], we next delineated the signaling activation profiles of RhoA/ROCK and PI3K/Akt pathways in the rat aortic SMCs responding to cyclic mechanical stretch. The SMCs grown on FN-coated silicone elastomer chambers and under given periods of stretching exposure were subjected to Western blotting detection (Figure 5C,D). The results pointed out that cyclic mechanical strains significantly triggered hyperphosphorylation of not only MBS, a downstream target of RhoA/ROCK, but also Akt, peaking at 15 and 30 post stretch min, respectively.

### Crosstalk of RhoA/ROCK with PI3K pathway and its involvement in stretch-induced HDGF up-regulation in rat aortic SMCs

To further clarify the role of the mechanical stretch-activated RhoA/ROCK and PI3K/Akt signal transduction in HDGF up-regulation in rat aortic SMCs, the cells were pretreated with either wortmannin, a selective PI3K inhibitor, or fasudil, a specific RhoA/ROCK blocker, and then underwent the consecutive and cyclic mechanical stretching procedures. Western blotting and densitometrical analysis data revealed that the mechanical stretch-induced RhoA/ROCK activity appeared to be an upstream mediator responsible for Akt phosphorylation and HDGF up-regulation in SMCs (Figure 5E,F).

### Involvement of RhoA/ROCK and PI3K/Akt signaling pathways in stretch-increased cytokine production in rat aortic SMCs

**Figure 6:**
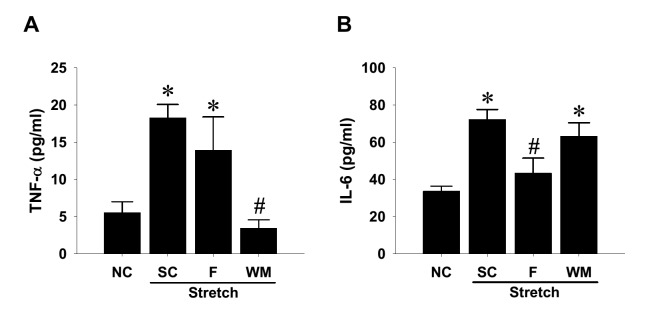
Involvement of signaling pathways in cyclic mechanical stretch-increased cytokine production in rat aortic smooth muscle cells (SMCs) SMCs were seeded on fibronectin-coated silicone elastomer chambers till full attachment. The cells were treated with selective kinase inhibitors at 10 μM or 0.1% DMSO solvent control (SC) for 1 h, followed by uniaxial and cyclic 10% stretches at constant frequency (1 Hz) for 24 h. Conditioned media from the stretched SMCs and nonstretching control (NC) were collected after consecutive 24-h stretching and subjected to ELISA detectionof TNF-α (**A**) and IL-6 (**B**). Data are shown in mean ± SEM; *P<0.05 vs. NC; ^#^*P*<0.05 vs. SC.

Because biomechanical stress stimulates IL-6 expression in mouse aortic SMCs [38], whereas the IL-6 content in human aortic wall is recently claimed to generate protective effect against acute vascular injury [39], we hence determined whether mechanical stretch-activated RhoA/ROCK and PI3K/Akt signaling cascades participate in the stretch-induced cytokine production in aortic SMCs. ELISA detection of conditioned media demonstrated significant elevation of both TNF-α and IL-6 cytokine production in SMCs (Figure 6). Blockade of PI3K activity dramatically suppressed the stretch-induced TNF-α cytokine release (Figure 6A), whereas inhibition of RhoA/ROCK activity significantly attenuated the overproduction of IL-6 stimulated by cyclic mechanical stretch (Figure 6B).

### Effect of *HDGF* gene silencing on constitutive cytokine production in rat aortic SMCs

**Figure 7:**
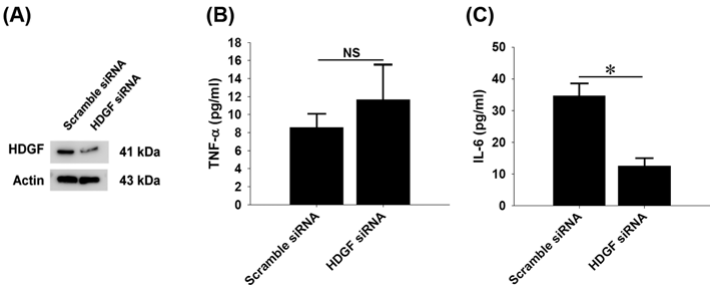
Effect of *HDGF* gene-silencing on the constitutive cytokine production in rat aortic smooth muscle cells (SMCs) Cultured SMCs received scramble nucleotides or *HDGF* siRNA for 24 h, followed by another 24-h incubation. (**A**) Western blotting data confirmed gene-silencing efficiency of siNRA-mediated *HDGF* gene knockdown after 48-h treatment. Twenty-four hours of conditioned media after *HDGF* gene knockdown were subjected to ELISA, revealing that *HDGF* gene modification did not affect TNF-α release (**B**), but significantly reduced IL-6 production (**C**) in SMCs. Data are shown in mean ± SEM; *P<0.05 compared between groups; NS, not significant.

To determine the significance of HDGF expression in constitutive expression of cytokine production in rat aortic SMCs, siRNA-mediated *HDGF* gene silencing were carried out and cellular lysates and supernatants were subjected to analyses of Western blot and ELISA respectively. *HDGF* gene knockdown was confirmed after 24-h siRNA delivery and another 24-h incubation (Figure 7A). The cytokine measurements of 24-h conditioned media by ELISA clearly showed that *HDGF* gene knockdown did not affect constitutive TNF-α (Figure 7B), but significantly suppressed IL-6 production in rat aortic SMCs (Figure 7C).

## Discussion

Vascular SMCs and endothelial cells present in blood vessel wall have the ability to respond to environmental stimuli and hemodynamic forces including radial stretch and shear stress. *In vitro* static cell culture has been arguable in that the conditions lacking physical factors may not reflect the full responsiveness of cultured cells, which frequently leads to discrepancy between *in vivo* and *in vitro* study results. The present study established an *in vitro* biomechanical instrumentation system, through which we examined the relationship between mechanical force and the HDGF expression in cultured rat aortic SMCs. We first characterized that ECM coating with FN provided a long-term cell adhesion durable for up to at least consecutive 24-h mechanical stretching and thus FN coating is preferentially suggested to be applied to studies involving SMCs. Whether FN coating is suitable for most of cell types awaits wider screening. The interaction between FN and specific integrin molecules, such as integrin α5β1, has been previously shown to provide stringent adhesion of vascular SMCs and contribute to arteriogenesis [40]. Supportive to this notion, mechanical stretch is previously demonstrated to regulate temporal and spatial expression FN and its cognate receptor integrin α5 in rat uterine SMCs [41]. The reproduced result in the present study supports that FN is a better ECM substratum inducing potent adhesion of cultured SMCs compared with the effect of type I collage (Figures 1 and 2). Furthermore, subsequent morphological observation showed that the uniaxial and cyclic mechanical stretch at constant frequency that mimics the vascular tone effect was found to induce SMC reorientation and realignment (Figure 3) as well as F-actin redistribution (Figure 4) after consecutive 6-h stretching. Consistent to previous studies, our data again confirmed the mechanical stretch-induced reorientation and realignment effects that have been previously reported in human bladder [4] and airway [5] SMCs as well as aortic SMCs derived from cows [6] and rats [7], illustrating the efficacy of the stretching instrumentation used in the present study.

In the context of the mechanical force-activated signal transduction in aortic SMCs, the present study demonstrated that the stretching action elicited signal activation in pathways, including RhoA/ROCK and PI3K/Akt cascades (Figure 5). Undoubtedly, the RhoA/ROCK activation has been known to govern multiple cellular activities, including cellular motility and contraction of SMCs [34]. The RhoA/ROCK-enhanced contractility of aortic vascular SMCs is closely associated with cytoskeletal assembly and organization [35,36], which is intriguingly correlated with the stretch-induced F-actin redistribution noted in the present study. Similar to previous observation [30], the present study demonstrated that cyclic mechanical strain also induced Akt activation in vascular SMCs. More importantly, the ELISA measurement suggest that the stretch-activated PI3K/Akt signaling is crucially related to the constitutive TNF-α, while the activated RhoA/ROCK activity is involved in constitutive IL-6 production in rat aortic SMCs (Figure 6). In fact, biomechanic stretch has been known to induce TNF-α overproduction in cardiovascular cell type such as cardiomyocytes [42,43], but this induction effect was not reported in all types of SMCs. Our results strongly implicate that the stretch-induced TNF-α up-regulation may additionally enhance synthesis of other proinflammatory cytokines and alter microenvironment of tissues under mechanical stress. The proinflammatory effect of increased TNF-α has been recently addressed to aggravate profibrogenic remodeling [44]. Conversely, stretching overload under hypertensive conditions reportedly induces clustering of TNF-α associated death receptors that cause apoptosis of not only cardiomyocytes [45] but also vascular SMCs [46]. In the context of the stretch-induced signaling, the regulatory role of PI3K in SMCs has been previously demonstrated in stretched bladder SMCs [47], monocyte chemotactic protein-3-treated human coronary SMCs [48], as well as TNF-α-exposed aortic SMCs [49]. All those studies, however, only focused on the involvement of PI3K signaling pathway activation in SMC proliferation. To the best of our knowledge, the present study is the first to report that the mechanical stretch-induced PI3K signaling is involved in TNF-α production in aortic SMCs. Similar to the stretch-induced inflammatory cytokines, cyclic stretches indeed increased *IL-6* mRNA in colonic SMCs [50] and IL-6 biosynthesis in aortic SMCs [38]. Our findings of kinase inhibition experiment further demonstrated the involvement of RhoA/ROCK signaling activity in the IL-6 production in aortic SMCs (Figure 6B). Although Deng et al. [51] reported that gene knockdown of ROCK1, a ROCK isoform, was found to reduce viability and inhibit proliferation of vascular SMCs, but it simultaneously up-regulated *de novo* IL-6 synthesis in arterial SMCs, suggesting a negative role of ROCK1 in IL-6 production. The seeming discrepancy in Deng’s and our data is very likely due to the existence of biomechanical factor in our system. However, this issue awaits further elucidation.

As proposed by earlier studies, HDGF has been regarded one of alarmins, which are highly expressed in and released from the cells under acute injury and convey the damage signal to immune system [52,53]. More intriguingly, the present study shows the first evidence that mechanical stretch indeed increased HDGF expression in aortic SMCs with concomitant PCNA up-regulation and histone H3 hyperphosphorylation (Figure 5A). On the contrary, siRNA-mediated *HDGF* gene silencing significantly lowered the constitutive IL-6 production in rat aortic SMCs (Figure 7). Based upon these findings, we hereby offer several implications for the possible roles of HDGF in vascular mechanics and pathophysiology. First, biomechanic force may induce and retain higher constitutive level of HDGF expression in aortic and/or vascular SMCs compared with the cells in static state. Due to the fact that the alignment rate of SMCs is dependent of cyclic straining frequency [7], we cannot rule out the possibility that the extent of HDGF up-regulation is also proportional to the cyclic frequency actually applied to the cells. Whether high frequency cyclic mechanical stretching induces higher levels of HDGF expression, however, remains further elucidation. Second, the endogenous expression of HDGF in aortic SMCs may play critical roles in regulation of cell proliferation, adhesion, and migration, thereby improving wound healing and arteriogenic processes during vascular injury. Third, the increased HDGF expression in stretched SMCs may serve as an upstream regulator that stimulates production of TNF-α and IL-6 cytokines in aortic and/or vascular SMCs. In addition to immunoregulatory function, the increased TNF-α production in abdominal aortic aneurismal wall is believed to stimulate aortic endothelial cell migration [54], whereas the increased IL-6 and subsequent STAT3 activation is more recently proposed to play a protective role in acute vascular injury [39]. The simultaneous HDGF and IL-6 up-regulation induced by cyclic mechanical stretch in cultured aortic SMCs raises the possibility that the up-regulated HDGF may benefit endovascular repair through activating IL-6/STAT3 axis. The role of HDGF in regulation of IL-6 biosynthesis in aortic SMCs might deserve to be studied in much depth.

In conclusion, the present study reproduced the reorienting and aligning effects of biomimetically mechanical stretch in cultured rat aortic SMCs and demonstrated that the cyclic mechanical stretch up-regulated HDGF expression and production of inflammatory cytokines, including TNF-α and IL-6 therein. The cyclic mechanical stretch may retain constitutive expression of HDGF responsible for regulation of physiological behaviors of SMCs and induce the cytokines contributing to reparation of vascular wall injury.

## Supporting information

**supplementary Figure SI:** Verification of mechanical stretch-induced deformation of silicone-based elastomer in this study. (A) The photograph of the silicone-based elastomer chamber pre-printed with 81 red dots in 9X9 format. The silicone chamber was mounted on the stretching device and received uni-axial and cyclic 20 % deformation at constant frequency (60 cycles/min). The chamber morphology was photographed under static (B) and stretched (C) conditions. The deformed area of red dots was morphometrically measured by using ImageJ analysis software and the results are shown as percentage change of dot area compared with original area (D). The overall deformation rate is 19.97±0.41%, while the deformation accuracy of the stretching device is 99.85%.

## Abbreviations

α-SMA: smooth muscle α-actin
ECM: extracellular matrix
FN: fibronectin
HDGF: hepatoma-derived growth factor
IL-6: interleukin-6
MAPK: mitogen activated protein kinase
PCNA: proliferative cell nuclear antigen
PI3K: phosphatidylinositol 3-kinase
SMC: smooth muscle cell
TNF-α: tumor necrosis factor-α
