# Supplementary material for: Cyclic mechanical stretch up-regulates hepatoma-derived growth factor expression in cultured rat aortic smooth muscle cells
Source: Biosci Rep. 2018 Mar 16;38(2):BSR20171398. doi: 10.1042/BSR20171398 (PMC5857908; doi:10.1042/BSR20171398)
Supplement: Supplementary file 1 [file bsr20171398_Supp1.pdf]

A B C

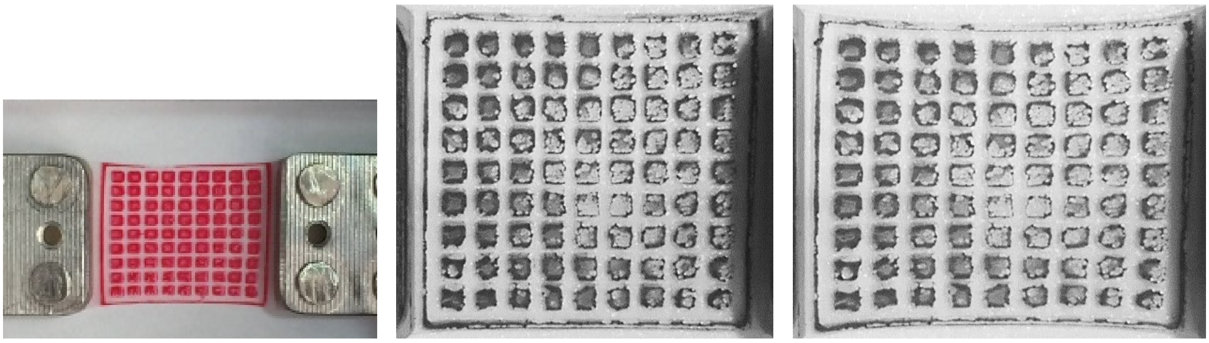

D

	1	2	3	4	5	6	7	8	9	
1	18.5%	18.1%	20.0%	19.8%	19.4%	20.1%	19.4%	19.9%	18.3%	19.3%
2	19.6%	19.8%	19.7%	19.7%	19.1%	19.9%	19.6%	20.5%	18.7%	19.6%
3	19.7%	20.3%	19.9%	19.8%	20.9%	21.9%	20.4%	20.0%	19.4%	20.3%
4	19.2%	20.8%	21.5%	22.0%	21.3%	21.4%	21.3%	19.2%	19.2%	20.6%
5	18.7%	20.7%	21.0%	19.8%	21.9%	21.8%	21.9%	19.2%	18.9%	20.4%
6	18.4%	19.5%	19.8%	20.3%	21.5%	19.3%	21.7%	20.4%	18.7%	20.0%
7	19.8%	21.3%	19.6%	19.8%	22.1%	19.6%	20.1%	19.8%	18.4%	20.1%
8	19.3%	19.6%	19.7%	19.6%	22.5%	19.2%	19.3%	20.7%	19.0%	19.9%
9	19.8%	18.4%	20.5%	18.6%	21.3%	20.3%	19.5%	18.2%	18.9%	19.5%

Supplementary Figure S1 Verification of mechanical stretch-induced deformation of silicone-based elastomer in this study. (A) The photograph of the silicone-based elastomer chamber pre-printed with 81 red dots in 9X9 format. The silicone chamber was mounted on the stretching device and received uni-axial and cyclic 20 % deformation at constant frequency (60 cycles/min). The chamber morphology was photographed under static (B) and stretched (C) conditions. The deformed area of red dots was morphometrically measured by using ImageJ analysis software and the results are shown as percentage change of dot area compared with original area (D). The overall deformation rate is 19.97±0.41%, while the deformation accuracy of the stretching device is 99.85%.
